# Supplementary material for: DMRforPairs: identifying Differentially Methylated Regions between unique samples using array based methylation profiles
Source: BMC Bioinformatics. 2014 May 15;15:141. doi: 10.1186/1471-2105-15-141 (PMC4046028; doi:10.1186/1471-2105-15-141)
Supplement: Additional file 2 — DMRforPairs output for the comparison of A431-MCF7 and NA17018-NA17105. Please start from the HTML files in each folder. Available via the BMC Bioinformatics website. [file 1471-2105-15-141-S2.zip › 1394847754114233_MOESM2_ESM/A431_MCF7/figures/15462.pdf]

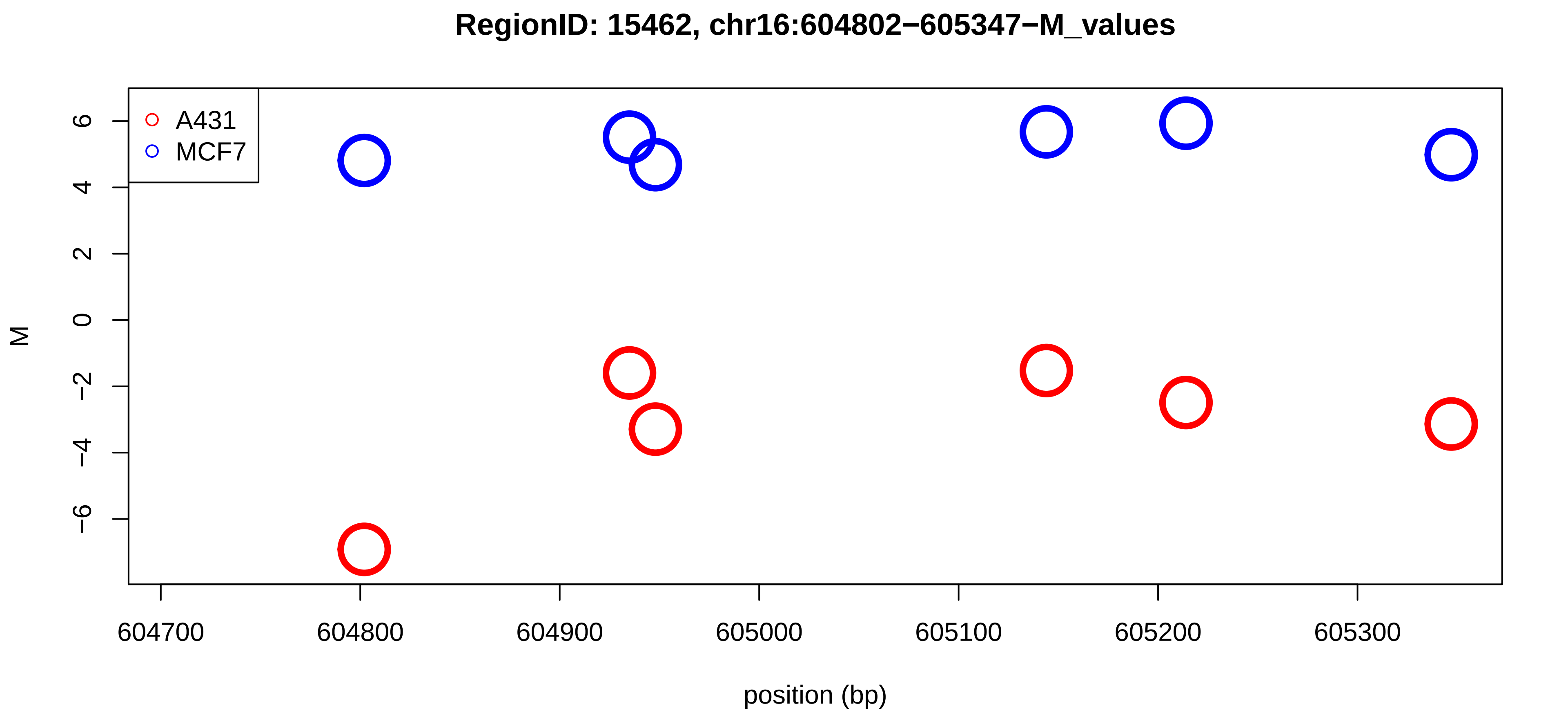

RegionID: 15462, chr16:604802−605347−M_values
A431
MCF7
M
6
4
2
0
−2
−4
−6
604700
604800
604900
605000
605100
605200
605300
position (bp)

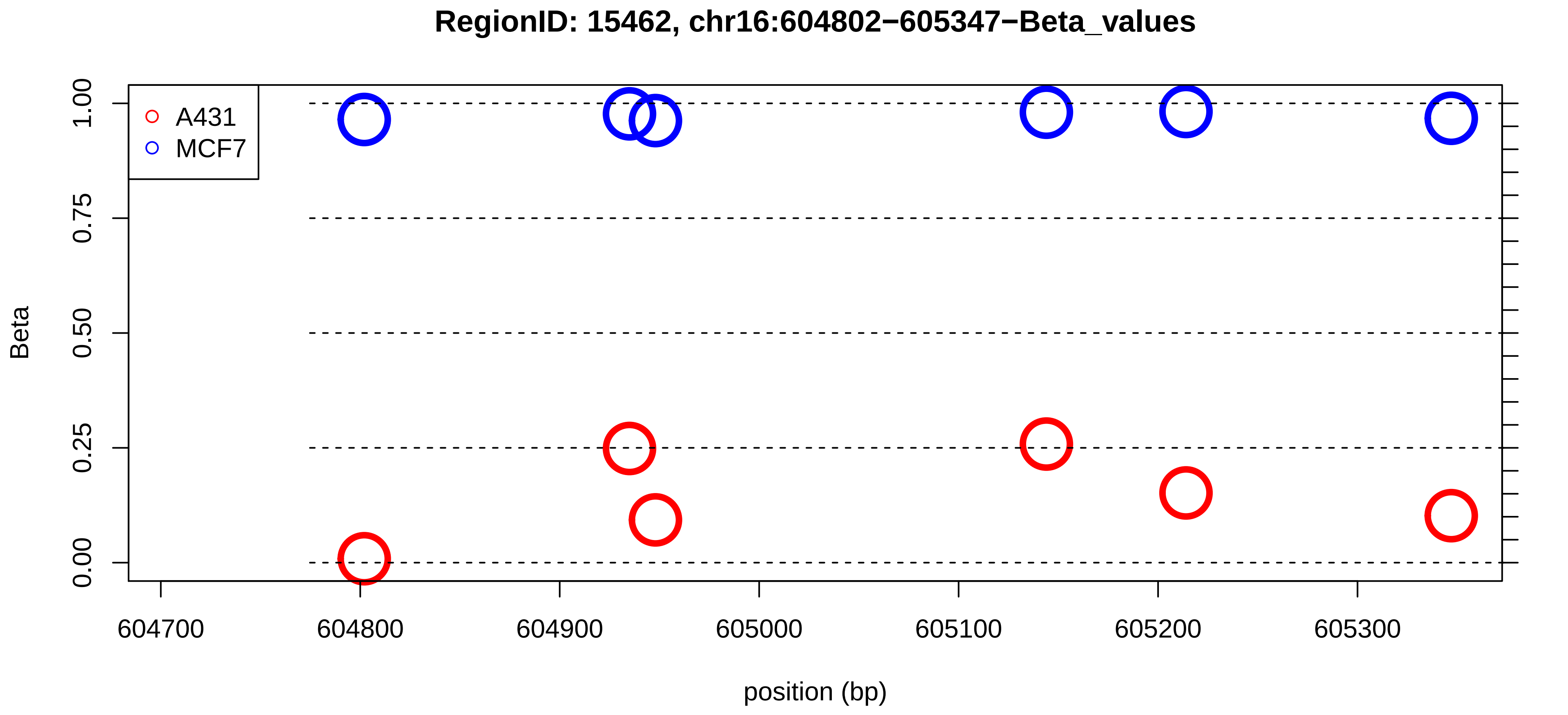

RegionID: 15462, chr16:604802−605347−Beta_values
A431
MCF7
Beta
1.00
0.75
0.50
0.25
0.00
604700
604800
604900
605000
605100
605200
605300
position (bp)

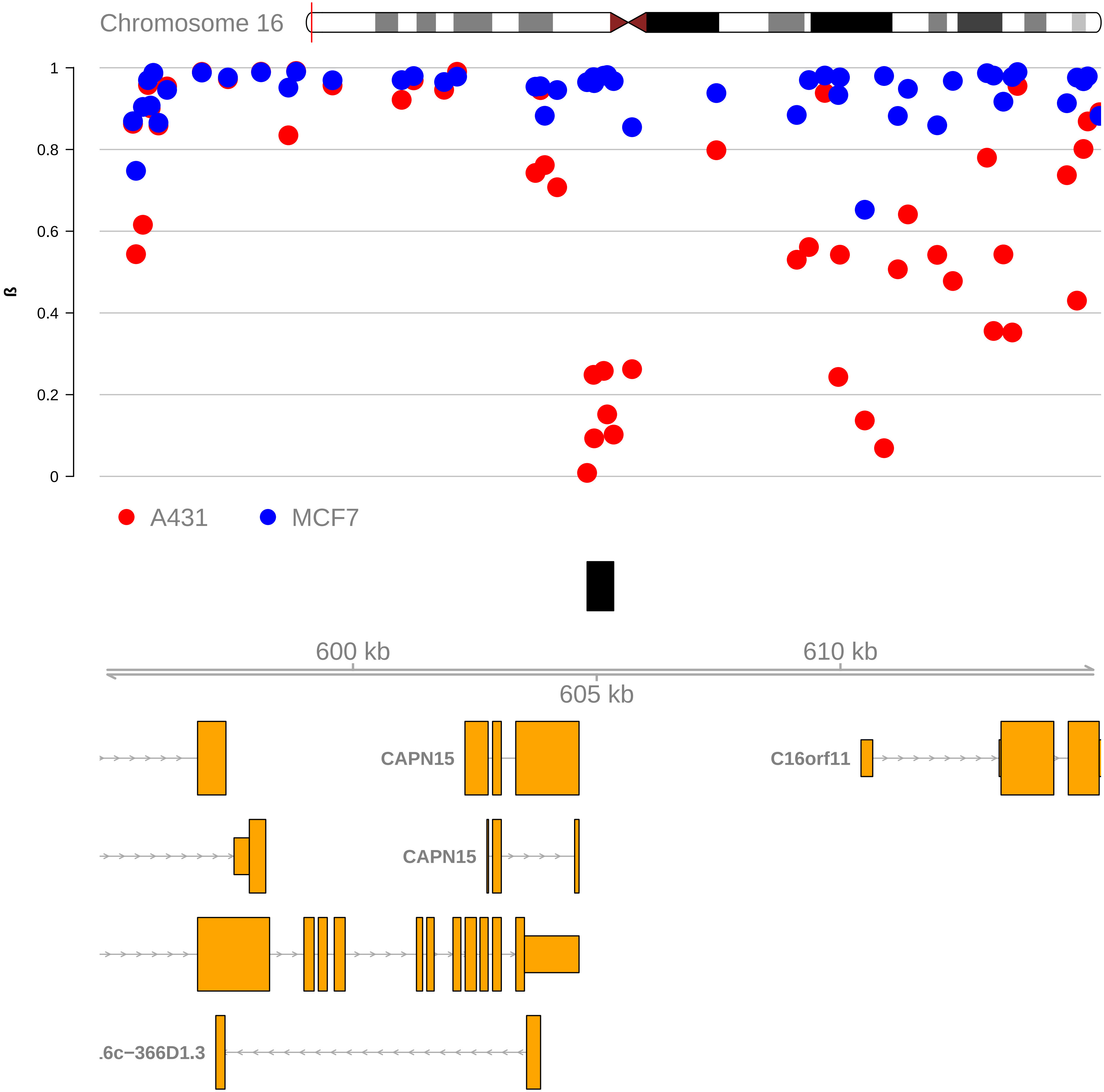

Chromosome 16
1
0.8
0.6
0.4
0.2
0
β
A431
MCF7
600 kb
610 kb
605 kb
CAPN15
C16orf11
CAPN15
.6c−366D1.3
